# Physical Activity, Sedentary Time and Physical Capability in Early Old Age: British Birth Cohort Study

**DOI:** 10.1371/journal.pone.0126465

**Published:** 2015-05-11

**Authors:** Andrew J. M. Cooper, Rebecca K. Simmons, Diana Kuh, Soren Brage, Rachel Cooper, NSHD scientific and data collection team

**Affiliations:** 1 MRC Epidemiology Unit, University of Cambridge, Institute of Metabolic Science, Box 285, Addenbrooke’s Hospital, Hills Road, Cambridge, CB2 0QQ, United Kingdom; 2 MRC Unit for Lifelong Health & Ageing at UCL, 33 Bedford Place, London, WC1B 5JU, United Kingdom; Universidad Pablo de Olavide, Centro Andaluz de Biología del Desarrollo-CSIC, SPAIN

## Abstract

**Purpose:**

To investigate the associations of time spent sedentary, in moderate-to-vigorous-intensity physical activity (MVPA) and physical activity energy expenditure (PAEE) with physical capability measures at age 60-64 years.

**Methods:**

Time spent sedentary and in MVPA and, PAEE were assessed using individually calibrated combined heart rate and movement sensing among 1727 participants from the MRC National Survey of Health and Development in England, Scotland and Wales as part of a detailed clinical assessment undertaken in 2006-2010. Multivariable linear regression models were used to examine the cross-sectional associations between standardised measures of each of these behavioural variables with grip strength, chair rise and timed up-&-go (TUG) speed and standing balance time.

**Results:**

Greater time spent in MVPA was associated with higher levels of physical capability; adjusted mean differences in each capability measure per 1standard deviation increase in MVPA time were: grip strength (0.477 kg, 95% confidence interval (CI): 0.015 to 0.939), chair rise speed (0.429 stands/min, 95% CI: 0.093 to 0.764), standing balance time (0.028 s, 95% CI: 0.003 to 0.053) and TUG speed (0.019 m/s, 95% CI: 0.011 to 0.026). In contrast, time spent sedentary was associated with lower grip strength (-0.540 kg, 95% CI: -1.013 to -0.066) and TUG speed (-0.011 m/s, 95% CI: -0.019 to -0.004). Associations for PAEE were similar to those for MVPA.

**Conclusion:**

Higher levels of MVPA and overall physical activity (PAEE) are associated with greater levels of physical capability whereas time spent sedentary is associated with lower levels of capability. Future intervention studies in older adults should focus on both the promotion of physical activity and reduction in time spent sedentary.

## Introduction

People aged 65 years or older represent one of the fastest growing segments of the worldwide population [1]. In the UK it is projected that the number of older adults will increase from 10.3 million in 2010, or 16.6% of the total population, to 17.0 million, or 23.2% of the population, by 2035 [2]. This will undoubtedly have considerable implications for the provision of health and social care within the National Health Service (NHS), where average spending is four to seven times higher than the average person in middle age [3]. One of the most salient consequences of the ageing process occurs as a result of decreases in muscle strength and physical performance, increasing the risk of disability, institutionalisation and mortality [4–7]. Though declines in physical capability are a natural consequence of age-related changes in the musculoskeletal system from midlife onwards, a large number of adults remain physically independent throughout later life.

Evidence from intervention and epidemiological studies has demonstrated that higher levels of physical activity are associated with better physical performance in mid-life and older age [8–16]. Time spent sedentary, which increases rapidly in old age, has also been highlighted as a potentially important risk factor predisposing to poor physical performance, independent of physical activity [17, 18]. Sedentary behaviours (e.g. extensive TV viewing) in mid-life have been associated with an increased risk of coronary heart disease morbidity [19] and mortality [20]. However, the relationship between objective measures of sedentary time and physical performance in later life is less clear. To our knowledge only two previous studies have examined these associations in older adults, but the sample sizes were small and important potential confounders such as cigarette smoking and education were not accounted for [21, 22].

In a population based prospective cohort study among 1727 adults we aimed to examine the associations of objectively monitored sedentary time and physical activity with measures of physical capability at 60–64 years. Physical capability measures of interest were grip strength, chair rises, standing balance and Timed Up-&-Go test, which are known to be important predictors for disability, institutionalisation and mortality in this age group [4–7].

## Materials and Methods

The Medical Research Council National Survey of Health and Development (MRC NSHD) is a socially stratified sample of 5362 singleton births (2547 males and 2815 females) that occurred in one week of March 1946 in mainland Britain, the design of which has been reported previously [23, 24]. In brief, between 2006 and 2010, when study participants were aged 60–64 years, 2856 eligible participants (those known to be alive and who had a known address in England, Scotland or Wales) were invited for an assessment at one of six clinical research facilities (CRFs) or to be visited by a research nurse at home. Invitations were not sent to those who had died (n = 778), were living abroad (n = 570), had previously withdrawn from the study (n = 594), or had been lost to follow-up (n = 564). Of those invited, 2229 (78%) were assessed, 1690 of whom attended a CRF while the remaining 539 received a home visit from a trained nurse. All participants gave written informed consent and the data collection at age 60–64 years was approved by the Greater Manchester Local Research Ethics Committee and the Scotland A Research Ethics Committee. Bona fide researchers can apply to access the NSHD data via a standard application procedure (further details available at: http://www.nshd.mrc.ac.uk/data.aspx).

### Assessment of physical activity and sedentary time

At the end of the clinical assessment, participants were invited to have their habitual physical activity assessed using a combined heart rate and movement monitor (Actiheart, CamNtech, Cambridge, UK) worn continuously for five days (recorded in 30-second resolution) [25]. Details of this assessment and the data derived are provided elsewhere [26], and are summarised here. Participants who attended a CRF, and who were willing and able, underwent a step test to individually calibrate heart rate [27]. For participants who did not complete an individual calibration test (those who were seen at their home or who did not undertake a step test, n = 723), we used all valid calibration tests to derive an age, sex, beta-blocker and sleeping heart rate adjusted group calibration equation for the translation of heart-rate into activity intensity. Heart rate data collected during the free-living period were processed using noise classification followed by Gaussian robust regression [28] and average activity intensity (J/min^/^kg) was estimated using a branched equation framework [29]. Resulting time-series data were summarised into physical activity energy expenditure (PAEE; kJ/kg^/^day), sedentary time (hours/day) and total volume of moderate-to-vigorous-intensity physical activity (MVPA, minutes/day), whilst minimising diurnal information bias caused by non-wear periods (segments of non-physiological data). Sedentary time was defined as a MET value (metabolic equivalent of task) of <1.5 in accordance with current convention [30] and MVPA as ≥3.0 METs, both using an individualised estimate of resting metabolic rate (RMR) to define one MET [31]. Despite being correlated, we chose a priori to investigate both PAEE and MVPA in our analyses because PAEE provides a measure of the total volume of activity; it represents all energy expended above resting and therefore includes activities spent at lower intensities where most daily activities occur, in particular in older populations [32, 33]. For example, we have previously shown that 68% of PAEE in this population is accumulated in the 1.5–3.0 MET intensity range [33]. In contrast, MVPA is a time domain measure which confers information about the intensity of the physical activity distribution; it represents the time spent above moderate intensity which would include activities such as walking, cycling, and other exercise, therefore allowing for the importance of more strenuous but also far less common activities to be directly estimated.

### Assessment of strength and physical performance

Assessments of grip strength, chair rise time, standing balance time and Timed Up-&-Go (TUG) were undertaken by nurses following standard protocols as described in detail elsewhere [34]. In brief, grip strength (kg) was measured isometrically using a Nottingham electronic handgrip dynamometer. The dynamometers were calibrated at the start of testing using a back-loading rig and are accurate, linear, and stable to ±0.5 kg [35]. The intra-participant retest variability for maximal voluntary tests of strength in those unused to such measurements is approximately ±9% [36]. Each participant made three attempts for each hand and the maximum of all six tests was used for analyses. Chair rise time (seconds) was measured as the time taken to rise from a sitting to a standing position with straight back and legs and to sit down again as fast as possible 10 times. For these analyses, chair rise performance is expressed as speed, i.e. number of chair rises in one minute, calculated as (10/measured chair rise time (s))*60. Standing balance time was measured as the longest time, up to a maximum of 30 seconds, that participants could maintain a one-legged stand with their eyes closed. Because the distribution of standing balance times were positively skewed, values were normalised using a natural logarithmic transformation (ln(seconds)) after addition of 1 to prevent negative values after transformation. TUG time was assessed as the time taken (seconds) to get up from a chair, walk 3 metres, turn around, walk back 3 metres and sit back in the chair at the participant’s normal, habitual pace; participants were allowed to use a walking aid to complete this test (n = 17). For these analyses TUG time is expressed as a measure of speed in metres/second.

### Covariates

Assessment of participants included physiological and anthropometric measures taken by trained nurses following standardised protocols, and completion of self-report questionnaires. Height (m) and weight (kg) were measured at 60–64 years using a fixed Stadiometer and Tanita electronic scales without shoes and in light clothing, respectively. Highest educational level attained by the age of 26 years was coded as: 1) degree or higher; 2) A levels, usually attained at 18 years, or their equivalents; 3) O levels, usually attained at 16 years, or their equivalents; 4) certificate of secondary education, clerical course, or equivalent, and 5) none. Occupational class at the age of 53 years (used to reflect main adult occupational status) was categorised using the Registrar General’s Social Classification into three groups: I or II (higher managerial, administrative and professional); III non-manual or III manual (intermediate), and IV or V (routine and manual). Smoking status at 60–64 years was categorised into current, former, or never. The presence of any long-term illness, health problem or disability which limited activities was self-reported using a question from the 2001 England and Wales Census (yes, no). Participants reported use of beta-blocker medication (yes, no).

### Exclusions

Of the 2229 participants assessed, we excluded participants who were unable or unwilling to wear a monitor for the assessment period and those who had insufficient valid data to derive habitual measures of physical activity and sedentary time (i.e. <2 days of monitor wear time) (n = 502). After exclusions, 1727 individuals had exposure data and a minimum of one outcome measure and so were included in these analyses.

### Statistical analysis

Firstly, to enable comparison of effect estimates across exposures we constructed standard deviation (SD) scores ((observed value—mean)/SD) for the continuous measures of sedentary time, MVPA and PAEE to analyse their linear associations with each outcome: grip strength, chair rise speed, standing balance time and TUG speed. In initial models (sex adjusted only) we formally tested for departure from linearity by categorising the specific SD scores for sedentary time, MVPA and PAEE into ten categories, refitting the model including dummy variables for each category, and performing a joint Wald test for the added parameters [37]. We found no evidence that the assumption of linearity had been violated (joint Wald test p-values all ≥0.09). To maintain the representativeness of the study population and consistency with previous analyses in this cohort [5], we assigned the mean of the lowest sex-specific quintile for grip strength, chair rise speed, standing balance time and TUG speed to participants who were unable to perform the specific test for health reasons (n = 34, n = 93, n = 62 and n = 20, respectively).

In initial linear regression models we tested for effect modification by sex by entering cross-product terms (i.e. MVPA*sex) with main effects for each exposure-outcome combination. No evidence of interaction by sex was found (all p-values ≥0.05). We subsequently constructed three different multivariable linear regression models to examine the associations of sedentary time, MVPA and PAEE with each outcome. Estimates were first adjusted for sex (model 1), and then further adjusted for height and weight to account for the known effects of body size on measures of strength and performance (model 2). In the final model (model 3) we additionally adjusted for education level, occupational class, smoking status and presence of long-term limiting illness or disability. Confounders were selected on the basis of previously published associations in the literature, biological reasoning and statistical associations between exposures and outcomes in the current dataset. To maintain statistical power and minimise potential for bias due to missing data, we imputed missing data for covariates (height n = 3, weight n = 3, education level n = 90, occupational class n = 8, smoking status n = 149 and presence of long-term limiting illness or disability n = 4) in the sample of 1727 participants with complete data on physical activity measures and at least one of the physical capability measures using the multiple imputation by chained equations method. Imputation models were run for each exposure—outcome relationship for the main confounder model using 10 multiply imputed datasets and Rubin’s combination rules to combine datasets. The predictor variables included in each of the imputation models were the outcome of interest and those included in the main confounder model.

We ran sensitivity analyses in which we: (1) excluded participants who did not undergo a step test for individual level calibration (up to n = 889); (2) excluded participants taking beta-blocker medications (n = 124); (3) used a more stringent definition of MVPA (≥3.5 METs) as opposed to an MVPA cut point of ≥3.0 METs; (4) examined all associations using the complete-case analysis method (that is, including only those participants with complete data for exposure, outcome and covariates). Finally, to enable comparison with previous studies, we also ran model 3 with additional adjustment for MVPA when examining associations of sedentary time and vice versa.

All statistical analyses were performed using Stata/SE 13.1 (Stata-Corp., TX, USA).

## Results

### Participant characteristics

The study sample of 1727 participants (defined as those with data on objectively measured physical activity and at least one physical performance measure) had a roughly equal distribution of men and women (48.5% and 51.5%, respectively), with a mean age of 63.3 (range: 60.3–64.9) years. Compared with participants included in the analyses, those excluded because of insufficient valid data to derive habitual measures of physical activity and sedentary time (n = 502) were more likely to have retired (45.9% compared with 50.2%, respectively); no other differences in socio-demographic characteristics were found. In comparison with those who underwent a step test to individually calibrate heart rate, those who were unable to undertake the step test had a significantly higher weight, were more likely to be current smokers and had lower levels of physical capability.

As shown in Table 1, the study sample of men and women performed similarly for each of the physical performance tests, except men had markedly higher grip strength than women. Men spent a greater proportion of the day in MVPA and had higher levels of PAEE than women.

**Table 1:** Characteristics of the study sample of 1727 participants from the MRC National Survey of Health and Development at age 60–64.

	Men (n = 837)	Women (n = 890)
**Age** (y)	63.2 (1.1)	63.3 (1.0)
**Strength and physical performance** ^**a**^		
Grip strength (kg)	46.4 (11.5)	27.0 (7.5)
Chair rise speed (stands/min)	26.2 (7.3)	24.9 (7.3)
Standing balance time (ln/s)	1.6 (0.6)	1.6 (0.5)
TUG speed (m/s)	0.7 (0.2)	0.7 (0.1)
**Sedentary time and physical activity**		
Time spent sedentary (hours/day; including sleep time)	17.4 (2.2)	17.3 (2.0)
Time spent in MVPA (min/day)	90.5 (64.9)	79.9 (54.9)
PAEE (kJ/kg/day)	38.1 (15.7)	34.2 (13.3)
**Anthropometry** ^**b**^		
Height (cm)	174.8 (6.6)	161.7 (6.0)
Weight (kg)	85.0 (13.6)	73.2 (14.8)
**Education level** ^**c**^		
Degree or higher	134 (16.9)	54 (6.4)
A levels or equivalent	354 (44.7)	460 (54.4)
GCE, O levels or equivalent	46 (5.8)	77 (9.1)
None	258 (32.6)	254 (30.1)
**Occupational class** ^**d**^		
Higher	462 (55.5)	355 (40.1)
Intermediate	293 (35.2)	378 (42.7)
Manual	78 (9.4)	153 (17.3)
**Smoking status** ^**e**^		
Never	226 (29.7)	294 (36.0)
Ex-smoker	460 (60.5)	430 (52.7)
Current	75 (9.9)	93 (11.4)
**Long-term limiting illness or disability** ^**f**^		
No	650 (77.8)	665 (74.9)
Yes	185 (22.2)	223 (25.1)

Data are means (SD) and *n* (%).

^a^Grip strength: men = 793, women = 819; chair rise speed: men = 785, women = 832; standing balance time: men = 803, women = 848; TUG time: men = 765, women = 824;

^b^Height: n = 834 for men; weight: n = 835 for men and n = 889 for women;

^c^Men = 792 and women = 845;

^d^Men = 833 and women = 886;

^e^Men = 761 and women = 817;

^f^Men = 835 and women = 888.

Note: Sedentary time was defined as a MET value of <1.5 in accordance with current convention [30] and MVPA as ≥3.0 METs using an individualised estimate of RMR to define one MET [31].

Sedentary time was strongly inversely correlated with MVPA (r = -0.70) and PAEE (r = -0.88). MVPA was strongly correlated with PAEE (r = 0.89). The median duration of valid combined heart rate and movement sensing data was 5.03 days (IQR: 4.8, 5.2 days).

### Sedentary time

Table 2 shows that in sex-adjusted models, one standard deviation score greater time spent sedentary was associated with lower grip strength (-0.588 kg, 95% confidence interval -1.062 to -0.115 kg), chair rise speed (-0.550 stands/min, -0.898 to -0.201 stands/min), standing balance time (-0.050 s, -0.076 to -0.024 s) and TUG speed (-0.021 m/s, -0.028 to -0.013 m/s). These effect estimates remained similar after additional adjustment for height, weight and other potential confounders in model 3, except for the association with chair rise speed which was substantially attenuated (-0.084 stands/min, -0.426 to 0.257 stands/min), largely owing to adjustment for long-term limiting illness or disability.

**Table 2:** Differences in mean levels of physical capability (95% confidence intervals) per one standard deviation difference in sedentary time, moderate-to-vigorous physical activity and physical activity energy expenditure at age 60–64 years using multivariable linear regression models.

	Sedentary (per 1 SD difference/day)*			MVPA (per 1 SD difference/day)*			PAEE (per 1 SD difference/day)*		
	β (95% CI)	β (95% CI)	β (95% CI)	β (95% CI)	β (95% CI)	β (95% CI)	β (95% CI)	β (95% CI)	β (95% CI)
	Model 1	Model 2	Model 3	Model 1	Model 2	Model 3	Model 1	Model 2	Model 3
**Difference in mean grip strength (kg)**	**-0.588(-1.062, -0.115)**	**-0.649(-1.114, -0.184)**	**-0.540(-1.013, -0.066)**	**0.638(0.166, 1.110)**	**0.599(0.143, 1.056)**	**0.477(0.015, 0.939)**	**0.632(0.158, 1.105)**	**0.753(0.282, 1.225)**	**0.634(0.156, 1.113)**
**Difference in mean chair rise speed (stands/min)**	**-0.550(-0.898, -0.201)**	-0.328(-0.677, 0.021)	-0.084(-0.426, 0.257)	**0.670(0.321, 1.018)**	**0.688(0.346, 1.031)**	**0.429(0.093, 0.764)**	**0.943(0.594, 1.292)**	**0.658(0.303, 1.013)**	**0.392(0.044, 0.739)**
**Difference in mean (ln) standing balance time (s)**	**-0.050(-0.076, -0.024)**	**-0.031(-0.056, -0.005)**	-0.024(-0.050, 0.002)	**0.036(0.010, 0.062)**	**0.037(0.012, 0.062)**	**0.028(0.003, 0.053)**	**0.073(0.047, 0.099)**	**0.047(0.021, 0.073)**	**0.039(0.012, 0.065)**
**Difference in mean TUG speed (m/s)**	**-0.021(-0.028, -0.013)**	**-0.016(-0.023, -0.008)**	**-0.011(-0.019, -0.004)**	**0.023(0.016, 0.031)**	**0.023(0.016, 0.031)**	**0.019(0.011, 0.026)**	**0.029(0.021, 0.036)**	**0.022(0.015, 0.030)**	**0.018(0.010, 0.025)**

Model 1: adjusted for sex.

Model 2: adjusted for sex, height and weight.

Model 3: adjusted for sex, height, weight, education level, occupational class, smoking status and long-term limiting illness or disability.

n = 1,646 for grip strength; n = 1,710 for chair rise speed; n = 1,713 for standing balance time and n = 1,609 for TUG speed.

Associations highlighted in bold are statistically significant at p<0.05

* Each one unit (standard deviation) change equates to: 2.1 hours/day difference in time spent sedentary; a 60 min/day difference in moderate-to-vigorous physical activity and a 14.7 kJ/kg/day difference in physical activity energy expenditure.

Effect estimates are from analyses using the multiple imputation by chained equations method run across 10 imputed datasets and using Rubin’s combination rules to combine datasets.

Definitions: Sedentary time was defined as a MET value of <1.5 in accordance with current convention [30] and MVPA as ≥3.0 METs using an individualised estimate of RMR to define one MET [31].

### MVPA

In the confounder model one standard deviation score greater time spent in MVPA was associated with better grip strength (0.477 kg, 0.015 kg to 0.939), chair rise speed (0.429 stands/min, 0.093 stands/min to 0.764 stands/min), standing balance time (0.028 s, 0.003 s to 0.053 s) and TUG speed (0.019 m/s, 0.011 m/s to 0.026 m/s).

### PAEE

Higher levels of PAEE were associated with better grip strength (0.634 kg, 0.156 kg to 1.113 kg), chair rise speed (0.392 stands/min, 0.044 stands/min to 0.739 stands/min), standing balance time (0.039 s, 0.012 s to 0.065 s) and TUG speed (0.018 m/s, 0.010 m/s to 0.025 m/s). The association between PAEE and grip strength was similar in the sex only adjusted model (0.632 kg, 95% confidence interval 0.158 kg to 1.105 kg) in comparison with the confounder model. The associations were strongest for chair rise speed, standing balance time and TUG speed in the sex plus height & weight models in comparison with the confounder models.

### Sensitivity analyses

Our findings remained unchanged when we excluded participants taking beta-blocker medications (n = 154) and when we ran analyses using an MVPA cut point of ≥3.5 METs as opposed to ≥3.0 METs, except for the association with chair rises which was stronger with the higher MVPA cut point (0.562 stands/min, 0.227 stands/min to 0.898 stands/min). Our findings were attenuated when we excluded participants who did not undergo a step test for individual level calibration (n = 723). Our findings were also generally attenuated when we repeated all analyses using the complete case method (S1 Table—Complete-case analysis). When we examined the association between sedentary time with additional adjustment for MVPA, the associations with grip strength and TUG speed were attenuated and no longer statistically significant. In contrast, when we examined the associations of MVPA adjusted for time spent sedentary, the association with TUG speed remained largely unchanged (0.021 m/s, 0.011 m/s to 0.031 m/s) and was stronger for chair rise speed (0.740 stands/min, 0.266 stands/min to 1.214 stands/min). The associations between MVPA and grip strength and standing balance time were no longer statistically significant.

## Discussion

We have shown that older adults who spend more time in MVPA and who expend more energy due to physical activity (PAEE) perform better across a range of physical capability measures including grip strength, chair rise speed, standing balance and TUG. In contrast, those who spend more time sedentary tend to perform worse across the same measures. The majority of these associations were not explained by height, weight and other known important confounders.

An important strength of our study is the use of objective measures of sedentary time, physical activity and physical performance. Previous studies have typically assessed these exposures and/or outcomes using self-report methods [38, 39] which are prone to recall and social desirability bias. Another important strength of our study was inclusion of individuals in the analyses who were unable to perform all of the physical capability tests for health reasons and those missing data on covariates, thereby ensuring that our findings are generalizable. Further, as our findings were attenuated in sensitivity analyses when these groups were excluded this suggests that the level of bias introduced may also have been minimised through their inclusion in the main analyses. An additional notable strength is that we have been able to demonstrate that our findings are not only robust to the exclusion of participants taking beta-blocker medications, but they are also not affected by use of a higher MVPA cut point. Importantly, as our study population is relatively homogenous for age, this allowed us to examine all associations independent of the strong confounding effect of age. Finally, as the NSHD participants at age 60–64 years are similar to the wider population of UK adults aged 60–64 years in terms of important socio-demographic factors [23], our findings are likely generalizable. A limitation of our study is the cross-sectional study design, which excludes inference regarding the direction of causality of the observed associations. As such, we cannot exclude the possibility that those who have lower levels of physical capability are less able to be physically active, or indeed that relationships may be bidirectional. Nevertheless, physical activity interventions among older adults have been shown to improve standing balance, walking speed and chair rise performances [12], lending support to the hypothesis examined in the present analyses. Second, our measure of sedentary time also includes sleep and therefore we cannot exclude the possibility of confounding by sleep duration; specifically, the interpretation of MVPA coefficients in the sedentary time adjusted model is therefore the equivalent of exchanging light intensity time for MVPA time. Finally, we cannot exclude the possibility of residual confounding by poorly measured covariates or confounding by factors not included in our models.

We confirm and extend previous findings by demonstrating that objective measures of sedentary time and physical activity are associated with objective measures of physical performance among older adults. Using data on 6228 men and women aged 65 years from the English Longitudinal Study of Ageing (ELSE), Hamer and Stamatakis [18] recently demonstrated that individuals who watch over 6 hours/day of TV have significantly lower grip strength than those who report watching fewer than 2 hours/day, an observation generally in agreement with that of Keevil and colleagues [40]. Gennuso et al. [38] previously showed that objectively measured MVPA is positively associated with self-reported physical function based on a categorical summary score of eight activities of daily living (including walking, stepping, lifting/carrying and standing from a chair) among 1,914 adults aged ≥65 years (mean 74.6 ± 6.5 years) in the NHANES study.

To the best of our knowledge only two other studies have used objective measures of sedentary time and physical activity to examine associations with physical performance [21, 22]. In a small study of 217 adults aged 78 years, Davis et al. [22] found inverse associations between sedentary time, chair rise speed and standing balance performance. These associations remained after adjustment for MVPA. In a similarly small study of 312 Portuguese older adults aged 74 years, Santos et al. [21] showed that participants who spent more time sedentary performed worse for chair rise speed and the 8-foot up-&-go test, whereas those who spent more time in MVPA performed better on the chair rise test. In contrast to our findings however, associations for sedentary time were independent of MVPA. The question of whether poorer physical performance is caused by time spent sedentary or whether it is a consequence of too little physical activity therefore remains to be answered. Plausible explanations for the differences in results between our study and those of Davis and Santos [21, 22] include different adjustment strategies and differences in age between study participants. The method used to measure sedentary time and MVPA is also likely to play an important role. We used a 24-hour combined heart-rate and movement monitoring protocol whereas both Davis and Santos [21, 22] used awake-time only accelerometers (Actigraph GT1M). Use of different physical activity assessment methods means that the definitions of sedentary time and MVPA across studies may not be directly comparable; as we demonstrated in the current study, associations can be sensitive to the cut-point used to define MVPA. Future studies that specifically assess the equivalence of seemingly similar physical activity constructs across different measurement methods are therefore needed in order to enable meaningful comparison of study findings.

To put our findings in context, if the associations observed between physical activity and time spent sedentary were causally associated with physical performance, it is not unreasonable to suggest that such differences would be expected to be strongly related with mortality rates. For example, Cooper et al have previously demonstrated that a higher grip strength of just 1 kg is associated with a 3% reduction in rates of mortality [44], and with regard to walking speed, Studenski et al demonstrated that a higher gait speed of just 0.1 m/s was associated with an 8% reduction in rates of mortality [41].

That we observed attenuation of our results when we included only participants who underwent a step test for individual level calibration of heart rate (the ‘healthiest’ individuals) suggests that future interventions aimed at helping the least healthy individuals to become more active will likely produce the greatest benefit at the population level. Future studies will be needed to specifically characterise the dose-response relationship between physical activity and measures of physical performance, similar to what has been established for coronary heart disease and type 2 diabetes [42]. Further research is also needed to identify the most effective interventions for increasing physical activity levels and decreasing time spent sedentary in older adulthood, especially given the likely cost-effectiveness of physical activity interventions for improving overall health and well-being [43, 44].

Our findings show that greater duration of time spent in MVPA and higher energy expenditure from physical activity (PAEE) are associated with significant differences in physical capability among older adults, whereas time spent sedentary is associated with poorer physical capability. Further studies are needed to establish whether changes in habitual sedentary time and physical activity lead to changes in physical performance in older adults, and to investigate which factors likely determine physical activity levels and their maintenance or decline in older adulthood.

## Supporting Information

S1 Table Complete-case analysis. (DOCX)

## References

[1] World Health Organization. Global Health and Aging2011 [cited 2014 26th June]. Available from: http://www.who.int/ageing/publications/global_health.pdf.

[2] Office for National Statistics. 2010-based National Population Projections[cited 2014 26th June]. Available from: http://www.ons.gov.uk/ons/rel/npp/national-population-projections/2010-based-projections/index.html.

[3] Howse K. Health and social care for older people in the UK: a snapshot view (Working paper 607)2007 [cited 2013 1st May]; 2013(1st May). Available from: http://www.ageing.ox.ac.uk/files/workingpaper_607.pdf.

[4] Cooper R, Kuh D, Hardy R. Objectively measured physical capability levels and mortality: systematic review and meta-analysis. Bmj. 2010;341:c4467.(doi). 10.1136/bmj.c4467 20829298 PMC2938886

[5] Cooper R, Strand BH, Hardy R, Patel KV, Kuh D. Physical capability in mid-life and survival over 13 years of follow-up: British birth cohort study. Bmj. 2014;348:g2219 10.1136/bmj.g2219 24787359 PMC4004787

[6] Guralnik JM, Ferrucci L, Pieper CF, Leveille SG, Markides KS, Ostir GV, et al Lower extremity function and subsequent disability: consistency across studies, predictive models, and value of gait speed alone compared with the short physical performance battery. J Gerontol A Biol Sci Med Sci. 2000;55(4):M221–31. 10811152 10.1093/gerona/55.4.m221 PMC12149745

[7] Guralnik JM, Simonsick EM, Ferrucci L, Glynn RJ, Berkman LF, Blazer DG, et al A short physical performance battery assessing lower extremity function: association with self-reported disability and prediction of mortality and nursing home admission. J Gerontol. 1994;49(2):M85–94. 8126356 10.1093/geronj/49.2.m85

[8] Cress ME, Buchner DM, Questad KA, Esselman PC, deLateur BJ, Schwartz RS. Exercise: effects on physical functional performance in independent older adults. J Gerontol A Biol Sci Med Sci. 1999;54(5):M242–8. 10362007 10.1093/gerona/54.5.m242

[9] Hillsdon MM, Brunner EJ, Guralnik JM, Marmot MG. Prospective study of physical activity and physical function in early old age. Am J Prev Med. 2005;28(3):245–50. 15766611 10.1016/j.amepre.2004.12.008

[10] Lang IA, Guralnik JM, Melzer D. Physical activity in middle-aged adults reduces risks of functional impairment independent of its effect on weight. J Am Geriatr Soc. 2007;55(11):1836–41. 17979901 10.1111/j.1532-5415.2007.01426.x

[11] Martin HJ, Syddall HE, Dennison EM, Cooper C, Sayer AA. Relationship between customary physical activity, muscle strength and physical performance in older men and women: findings from the Hertfordshire Cohort Study. Age Ageing. 2008;37(5):589–93. 10.1093/ageing/afn148 Epub 2008 Jul 29. 18664518

[12] Pahor M, Blair SN, Espeland M, Fielding R, Gill TM, Guralnik JM, et al Effects of a physical activity intervention on measures of physical performance: Results of the lifestyle interventions and independence for Elders Pilot (LIFE-P) study. J Gerontol A Biol Sci Med Sci. 2006;61(11):1157–65. 17167156 10.1093/gerona/61.11.1157

[13] Patel KV, Coppin AK, Manini TM, Lauretani F, Bandinelli S, Ferrucci L, et al Midlife physical activity and mobility in older age: The InCHIANTI study. Am J Prev Med. 2006;31(3):217–24. Epub 2006 Aug 2. 16905032 10.1016/j.amepre.2006.05.005 PMC2646092

[14] Visser M, Pluijm SM, Stel VS, Bosscher RJ, Deeg DJ. Physical activity as a determinant of change in mobility performance: the Longitudinal Aging Study Amsterdam. J Am Geriatr Soc. 2002;50(11):1774–81. 12410894 10.1046/j.1532-5415.2002.50504.x

[15] Cooper R, Mishra GD, Kuh D. Physical activity across adulthood and physical performance in midlife: findings from a British birth cohort. Am J Prev Med. 2011;41(4):376–84. 10.1016/j.amepre.2011.06.035 21961464 PMC3185208

[16] Dodds R, Kuh D, Aihie Sayer A, Cooper R. Physical activity levels across adult life and grip strength in early old age: updating findings from a British birth cohort. Age Ageing. 2013;42(6):794–8. 10.1093/ageing/aft124 23981980 PMC3809720

[17] Seguin R, Lamonte M, Tinker L, Liu J, Woods N, Michael YL, et al Sedentary Behavior and Physical Function Decline in Older Women: Findings from the Women's Health Initiative. J Aging Res. 2012;2012:271589 10.1155/2012/271589 22675631 PMC3364591

[18] Hamer M, Stamatakis E. Screen-based sedentary behavior, physical activity, and muscle strength in the English longitudinal study of ageing. PLoS One. 2013;8(6). 10.1371/journal.pone.0066222 PMC3670922 23755302

[19] Wijndaele K, Brage S, Besson H, Khaw KT, Sharp SJ, Luben R, et al Television viewing and incident cardiovascular disease: prospective associations and mediation analysis in the EPIC Norfolk Study. PloS one. 2011;6(5):e20058 Epub 2011/06/08. 10.1371/journal.pone.0020058 21647437 PMC3102066

[20] Wijndaele K, Brage S, Besson H, Khaw KT, Sharp SJ, Luben R, et al Television viewing time independently predicts all-cause and cardiovascular mortality: the EPIC Norfolk study. Int J Epidemiol. 2011;40(1):150–9. 10.1093/ije/dyq105 Epub 2010 Jun 23. 20576628

[21] Santos DA, Silva AM, Baptista F, Santos R, Vale S, Mota J, et al Sedentary behavior and physical activity are independently related to functional fitness in older adults. Exp Gerontol. 2012;47(12):908–12. 10.1016/j.exger.2012.07.011 Epub Aug 1. 22884978

[22] Davis MG, Fox KR, Stathi A, Trayers T, Thompson J, Cooper AR. Objectively Measured Sedentary Time and Lower Extremity Function in Older Adults. J Aging Phys Act. 2013;1:1. 10.1123/japa.2013-0042 24085473

[23] Stafford M, Black S, Shah I, Hardy R, Pierce M, Richards M, et al Using a birth cohort to study ageing: representativeness and response rates in the National Survey of Health and Development. Eur J Ageing. 2013;10(2):145–57. 23637643 10.1007/s10433-013-0258-8 PMC3637651

[24] Kuh D, Pierce M, Adams J, Deanfield J, Ekelund U, Friberg P, et al Cohort profile: updating the cohort profile for the MRC National Survey of Health and Development: a new clinic-based data collection for ageing research. Int J Epidemiol. 2011;40(1):e1–9. 10.1093/ije/dyq231 21345808 PMC3043283

[25] Brage S, Brage N, Franks PW, Ekelund U, Wareham NJ. Reliability and validity of the combined heart rate and movement sensor Actiheart. European journal of clinical nutrition. 2005;59(4):561–70. Epub 2005/02/17. doi: 10.1038/sj.ejcn.1602118. 15714212 15714212 10.1038/sj.ejcn.1602118

[26] Espana-Romero V, Golubic R, Martin KR, Hardy R, Ekelund U, Kuh D, et al Comparison of the EPIC Physical Activity Questionnaire with combined heart rate and movement sensing in a nationally representative sample of older British adults. PLoS One. 2014;9(2):e87085 10.1371/journal.pone.0087085 eCollection 2014. 24516543 PMC3916297

[27] Brage S, Ekelund U, Brage N, Hennings MA, Froberg K, Franks PW, et al Hierarchy of individual calibration levels for heart rate and accelerometry to measure physical activity. J Appl Physiol. 2007;103(2):682–92. 17463305 10.1152/japplphysiol.00092.2006

[28] Stegle O, Fallert SV, MacKay DJ, Brage S. Gaussian process robust regression for noisy heart rate data. IEEE transactions on bio-medical engineering. 2008;55(9):2143–51. Epub 2008/08/21. 10.1109/tbme.2008.923118 . 18713683

[29] Brage S, Brage N, Franks PW, Ekelund U, Wong MY, Andersen LB, et al Branched equation modeling of simultaneous accelerometry and heart rate monitoring improves estimate of directly measured physical activity energy expenditure. J Appl Physiol. 2004;96(1):343–51. Epub 2003 Sep 12. 12972441 10.1152/japplphysiol.00703.2003

[30] Sedentary Behaviour Research Network. Letter to the Editor: Standardized use of the terms "sedentary" and "sedentary behaviours". Applied physiology, nutrition, and metabolism. 2012;37(3):540–2. Epub 2012/05/01. doi: 10.1139/h2012-024. 22540258 22540258 10.1139/h2012-024

[31] Henry CJ. Basal metabolic rate studies in humans: measurement and development of new equations. Public Health Nutr. 2005;8(7A):1133–52. 16277825 10.1079/phn2005801

[32] NHS Information Centre for Health and Social Care. Health Survey for England 2008: Physical Activity and Fitness. London: NHS. Information Centre for Health and Social Care 2009.

[33] Golubic R, Martin KR, Ekelund U, Hardy R, Kuh D, Wareham N, et al Levels of physical activity among a nationally representative sample of people in early old age: results of objective and self-reported assessments. Int J Behav Nutr Phys Act. 2014;11:58 10.1186/479-5868-11-58 24885497 PMC4038114

[34] Kuh D, Bassey EJ, Butterworth S, Hardy R, Wadsworth ME. Grip strength, postural control, and functional leg power in a representative cohort of British men and women: associations with physical activity, health status, and socioeconomic conditions. J Gerontol A Biol Sci Med Sci. 2005;60(2):224–31. 15814867 10.1093/gerona/60.2.224

[35] Kuh D, Hardy R, Butterworth S, Okell L, Wadsworth M, Cooper C, et al Developmental origins of midlife grip strength: findings from a birth cohort study. J Gerontol A Biol Sci Med Sci. 2006;61(7):702–6. 16870632 10.1093/gerona/61.7.702

[36] Bassey EJ. Longitudinal changes in selected physical capabilities: muscle strength, flexibility and body size. Age Ageing. 1998;27(Suppl 3):12–6. 10408678 10.1093/ageing/27.suppl_3.12

[37] Jann B. nlcheck: Stata module to check linearity assumption after model estimation. Available from http://ideas.repec.org/.2008 [cited 2014 10th June].

[38] Gennuso KP, Gangnon RE, Matthews CE, Thraen-Borowski KM, Colbert LH. Sedentary behavior, physical activity, and markers of health in older adults. Med Sci Sports Exerc. 2013;45(8):1493–500. 10.249/MSS.0b013e318288a1e5 23475142 PMC5764165

[39] Paterson DH, Warburton DE. Physical activity and functional limitations in older adults: a systematic review related to Canada's Physical Activity Guidelines. Int J Behav Nutr Phys Act. 2010;7:38 10.1186/479-5868-7-38 20459782 PMC2882898

[40] Keevil V, Wijndaele K, Luben R, Sayer AA, Wareham NJ, Khaw KT. Television Viewing, Walking Speed, and Grip Strength in a Prospective Cohort Study. Medicine & Science in Sports & Exercise. 2014;Published Ahead-of-Print. 10.1249/MSS.0000000000000453 PMC5642351 25785826

[41] Studenski S, Perera S, Patel K, Rosano C, Faulkner K, Inzitari M, et al Gait speed and survival in older adults. Jama. 2011;305(1):50–8. 10.1001/jama.2010.1923 21205966 PMC3080184

[42] Chief Medical Officers Of England Scotland Wales ANI. Start Active, Stay Active: A report on physical activity from the four home countries’ Chief Medical Officers2011 [cited 2014 10th July]. Available from: http://www.dh.gov.uk/en/Publicationsandstatistics/Publications/.

[43] Roux L, Pratt M, Tengs TO, Yore MM, Yanagawa TL, Van Den Bos J, et al Cost effectiveness of community-based physical activity interventions. Am J Prev Med. 2008;35(6):578–88. 10.1016/j.amepre.2008.06.040 19000846

[44] Munro J, Brazier J, Davey R, Nicholl J. Physical activity for the over-65s: could it be a cost-effective exercise for the NHS? J Public Health Med. 1997;19(4):397–402. 9467144 10.1093/oxfordjournals.pubmed.a024667

