# Supplementary material for: Physical Activity, Sedentary Time and Physical Capability in Early Old Age: British Birth Cohort Study
Source: PLoS One. 2015 May 11;10(5):e0126465. doi: 10.1371/journal.pone.0126465 (PMC4427100; doi:10.1371/journal.pone.0126465)
Supplement: S1 Table — (DOCX) [file pone.0126465.s001.docx]

**S1.** Differences in mean levels of physical capability (95% confidence intervals) per one standard deviation difference in sedentary time, moderate-to-vigorous physical activity and physical activity energy expenditure at age 60-64 years using multivariable linear regression models: a complete-case analysis

|  | **Sedentary (per 1 unit difference/day)*** | | | **MVPA (per 1 unit difference/day)*** | | | **PAEE (per 1 unit difference/day)*** | | |
| --- | --- | --- | --- | --- | --- | --- | --- | --- | --- |
|  | **β (95% CI)** | **β (95% CI)** | **β (95% CI)** | **β (95% CI)** | **β (95% CI)** | **β (95% CI)** | **β (95% CI)** | **β (95% CI)** | **β (95% CI)** |
|  | **Model 1** | **Model 2** | **Model 3** | **Model 1** | **Model 2** | **Model 3** | **Model 1** | **Model 2** | **Model 3** |
| **Difference in mean grip strength (kg)** | -0.274  (-0.783, 0.235) | -0.387  (-0.887, 0.112) | -0.341  (-0.851, 0.169) | **0.529**  **(0.026, 1.033)** | **0.540**  **(0.054, 1.025)** | **0.497**  **(0.004, 0.991)** | 0.397  (-0.107, 0.901) | **0.595**  **(0.092, 1.097)** | **0.554**  **(0.043, 1.065)** |
| **Difference in mean chair rise speed (stands/min)** | -0.257  (-0.630, 0.116) | -0.075  (-0.449, 0.299) | 0.034  (-0.336, 0.405) | **0.462**  **(0.099, 0.825)** | **0.467**  **(0.108, 0.826)** | 0.337  (-0.019, 0.692) | **0.643**  **(0.276, 1.010)** | **0.404**  **(0.029, 0.778)** | 0.276  (-0.094, 0.647) |
| **Difference in mean (ln) standing balance time (s)** | -0.026  (-0.055, 0.002) | -0.007  (-0.036, 0.021) | -0.006  (-0.034, 0.023) | 0.021  (-0.007, 0.049) | 0.021  (-0.006, 0.049) | 0.017  (-0.011, 0.045) | **0.051**  **(0.023, 0.079)** | 0.026  (-0.002, 0.055) | 0.023  (-0.006, 0.051) |
| **Difference in mean TUG speed**  **(m/s)** | **-0.017**  **(-0.025, -0.009)** | **-0.013**  **-0.021, -0.004)** | **-0.010**  **(-0.018, -0.002)** | **0.022**  **(0.014, 0.030)** | **0.022**  **(0.014, 0.030)** | **0.019**  **(0.011, 0.027)** | **0.026**  **(0.018, 0.034)** | **0.020**  **(0.012, 0.028)** | **0.017**  **(0.009, 0.025)** |
| Model 1: adjusted for sex.  Model 2: adjusted for sex, height and weight.  Model 3: adjusted for sex, height, weight, education level, occupational class, smoking status and long-term limiting illness or disability.  n=1,395 for grip strength; n=1,407 for chair rise speed; n=1,436 for standing balance time and n=1,375 for TUG speed.  Associations highlighted in bold are statistically significant at p<0.05  * Each one unit (standard deviation) change equates to: 2.1 hours/day difference in time spent sedentary; a 60 min/day difference in moderate-to-vigorous physical activity and a 14.7 kJ/kg/day difference in physical activity energy expenditure.  Definitions: sedentary time was defined as a MET value of <1.5 in accordance with current convention [[30](#_ENREF_30)] and MVPA as ≥3.0 METs using an individualised estimate of RMR to define one MET [[31](#_ENREF_31)] | | | | | | | | | |
